# Peer Relationship Profiles among Early Adolescents from Low-Income Families: The Unique and Combined Effects of Attachment to Mothers and Conscientiousness

**DOI:** 10.3390/ijerph20054349

**Published:** 2023-02-28

**Authors:** Xiaoyu Lan, Chen Wang, Guanyu Cui

**Affiliations:** 1 Promenta Research Center, Department of Psychology, University of Oslo, 0373 Oslo, Norway; 2 Center for Brain, Mind and Education, Shaoxing University, Shaoxing 312000, China; 3 Department of Psychology, School of Education, Wenzhou University, Wenzhou 325035, China; 4 Research Center for Psychology and Behavior, Wenzhou University, Wenzhou 325035, China

**Keywords:** peer relationships, attachment to mothers, conscientiousness, low-income families, early adolescents, person-centered approach

## Abstract

Using research data gathered from multiple sources, the current study explored positive aspects of peer relationship profiles (indexed by peer-nominated acceptance and self-reported friendships) in a person-centered approach among early adolescents from low-income families. Moreover, this study investigated the unique and combined associations of adolescents’ attachment to mothers and parent-rated conscientiousness with emerging peer relationship profiles. A total of 295 early adolescents (42.7% girls; *M_age_* = 10.94, *SD* = 0.80) were involved in this study. Latent profile analysis identified three empirically derived peer relationship profiles: “isolated” (14.6%), “socially competent” (16.3%), and “average” (69.1%). Moderation analyses further showed that adolescents with secure attachment to mothers tend to have group memberships in socially competent and average profiles than the isolated profile. Such an association pattern was more heightened for those with higher conscientiousness (versus lower conscientiousness).

## 1. Introduction

Over recent decades, the world has been experiencing rapid modernization and urbanization, but alongside this dramatic development, concerns have emerged about the well-being of adolescents from low-income families [1,2]. Adolescents with this economically disadvantaged family background are exposed to various socio-contextual risk factors that hinder healthy development [3,4]. Supportive peer interactions in this regard play a crucial role in the adaptive patterns of adolescents from low-income families.

Yet, despite increasing awareness of adolescents from low-income families, surprisingly little research has been conducted on early adolescence, a developmental period with multiple biological, psychological, and social transitions and challenges [5,6,7]. Those challenges may accumulate to exacerbate risk in the context of low-income families [8]. Meanwhile, during early adolescence, a desire for autonomy and the growing amount of time spent at school highlight the importance for adolescents to maintain successful peer interactions [9,10,11]. Positive peer interactions during this life period provide not only a sense of emotional security and reliable alliance, but also a forum where social cohesion and competence can develop [12]. Researchers should therefore investigate the environmental and personal correlates of peer relationships in early adolescence and gain professional knowledge on facilitating successful social integration and ultimately improving the well-being of young adolescents from low-income families.

### 1.1. A Person-Centered Approach to Peer Relationships

Peer relationships, as a broad term, encompass a few interrelated but partially distinct constructs [13,14]. In the current study, we focused on positive aspects of peer relationships—peer acceptance and friendships. Specifically, peer acceptance, which indexes the group status of peer relationships, refers to the degree to which a child is liked by his or her peers [13,15]. By contrast, friendships describe dyadic and reciprocated liking among peers, given that they often exhibit mutual trust and loyalty to each other and engage in mutually beneficial social exchanges [13]. Past research has typically focused on a single aspect of peer relationships, either peer acceptance or friendships. Having a comprehensive picture of early adolescents’ peer relationships, however, requires simultaneously incorporating both the group- and dyadic-level factors.

Further, past research has relied predominantly on a traditional variable-centered approach (e.g., correlations, regressions, and path analyses), generating a limited scope of adolescents’ social adjustment. Such an approach often assumes homogeneity in the population, obscuring the fact that multiple aspects of peer relationships do not operate independently but rather as part of an integrated system within the individuals [16,17,18]. In response to this methodological limitation, the present research used a person-centered approach (i.e., a latent profile analysis) to identify peer relationship profiles, in which we classified adolescents based on similar values on different indicators of peer relationships (e.g., peer acceptance and friendships). According to the existing limited literature on peer relationship profiles, Chen et al. (2019) found the following four empirically derived peer relationship patterns among Chinese adolescents based on two aspects of peer relationships (i.e., peer acceptance and friendships): “socially competent (high scores on both peer acceptance and friendships)”, “socially accepted (high scores on peer acceptance but low scores on friendships)”, “isolated (low scores on both peer acceptance and friendships)”, and “rejected (low scores on peer acceptance but high scores on friendships). [19]” Relationship patterns among early adolescents from low-income urban families, however, remain inadequately understood in terms of peer relationship patterns. In accordance with a person-centered approach, examining how peer acceptance and friendships characterize distinct peer relationship profiles among early adolescents from low-income families is both conceptually important and practically meaningful.

To investigate the correlates of peer relationships in early adolescence, we first regarded relationships with parents as the proximal context in which adolescents observe and form emotional ties and develop future interpersonal relationships [20,21,22]. From the perspective of both adolescents and parents, mothers are often regarded as the primary caregivers within the family, a notion particularly pronounced in Chinese society [23]. Following the spillover theory [20,21,24], youth carry relationship dynamics from one context (e.g., mother-child relationships) to the other (peer relationships). In addition to this proximal context, the optimal socioemotional functions are determined by ecological factors embedded in multiple environmental and personal layers [25]. The interaction between environmental influences and personal attributes, rather than the main effects, is the key to developmental processes [26,27,28]. To fully capture the conditional processes contributing to the social adjustment of adolescents from low-income contexts, an ignored personal characteristic—conscientiousness—should be considered. In the following sections, we review these two focal variables, starting with the presentation of attachment to mothers.

### 1.2. Attachment to Mothers

Attachment refers to an enduring emotional bond of children to their primary caregivers, such as mothers [29]. The attachment theory posits that the emotional bond established between the mother and a child serves as a schema from which the child’s later relationships are formed [30,31]. Adolescents who establish secure base relationships with their responsive and supportive mothers, for example, would learn a set of social skills and the reciprocal nature of social interactions, which adolescents can then apply to peer interactions [32,33]. Adolescents are then also more confident in exploring new relationships with peers [34,35]. Studying the relationships between attachment to mothers and peer relationships is particularly important for adolescents from low-income families. This is because secure and reliable emotional bonds between mothers and children can provide protection, comfort, and support, protecting youth from a myriad of adverse outcomes triggered by family poverty [31].

From an empirical research perspective, past work has exhibited a positive association between secure attachment to mothers and peer relationships. Granot and Mayseless (2012), for instance, found that secure mother-child attachment was positively related to prosocial orientation in early adolescents’ peer relationships [36]. The authors found that adolescents who experienced a secure mother-child attachment could generalize regulation strategies and social knowledge into the affiliative system. Despite the aforementioned theoretical and empirical supports, the relationships between attachment to mothers and peer relationships in early adolescents from low-income families are still underexplored. Further, some researchers have suggested that the strength of this association may depend on the presence versus absence of individual characteristics [37,38]. In the current study, we therefore extended prior scholarship by investigating the moderating role of conscientiousness therein.

### 1.3. Conscientiousness

Conscientiousness, as a personality trait, reflects behavioral tendencies involving orderliness, self-control, industriousness, and responsibility [39]. Conscientious individuals tend to follow socially prescribed norms and rules, effectively manage behaviors and emotions, work hard to achieve goals, and be responsible concerning social activities [40]. Past research has supported the incremental effect of conscientiousness on positive peer relations even after controlling for the other four personality traits according to the Big Five framework [41]. Little research has, however, focused on the role of conscientiousness among adolescents from low-come family backgrounds. This knowledge gap is striking because conscientiousness is strongly linked to positive interpersonal relationships and adaptive responses to stress [42,43].

Further, conscientiousness may not only directly affect youth peer relationships, but also may moderate the association between attachment to mothers and peer relationships. According to the extant research, there are two plausible interaction patterns (i.e., additive and compensatory). First, prior research has documented that personality may affect adolescents’ susceptibility to environmental influences, such as attachment to mothers [37,38]. Because conscientious adolescents are more likely to establish a close relationship with their mothers and are generally more aware of others, the positive association between secure attachment to mothers and positive peer relationships would be stronger for adolescents with higher conscientiousness (versus those with lower conscientiousness). Second, secure attachment to mothers and conscientiousness may serve as two compensatory mechanisms influencing peer relationships [44]. In this scenario, for adolescents with higher levels of conscientiousness, secure attachment to mothers is not positively related to peer relationships because conscientiousness works as a self-promoted motive to maintain positive peer relationships; whereas for adolescents with lower levels of conscientiousness, secure attachment to mothers is positively related to peer relationships because interpersonal skills learned from mother-child relationships can be generalized to developing and maintaining good peer relationships though these adolescents are themselves less able to manage behaviors and follow the rules. Considering that both interaction patterns have plausible justifications and empirical support, the present research would add to the extant research by further clarifying the interaction patterns between attachment to mothers and conscientiousness in peer relationships among early adolescents from low-income families.

In addition to the above conceptual gaps in the extant literature, prior studies on personality development have predominantly relied on adolescents’ self-reported personality traits, providing restricted information (high in missing values and difficult-to-judge accuracy; [45]). This limitation is particularly highlighted during early adolescence, a developmental period during which the personality is not completely mature [46]. Young adolescents may be unable to provide valid self-ratings of their personality traits, and parental ratings can therefore be adopted as reliable substitutes [47].

### 1.4. The Present Study

Using research data gathered from multiple sources, the present study aimed to explore positive aspects of peer relationship profiles (indexed by peer-nominated acceptance and self-reported friendships) among early adolescents from low-income urban families. This study also investigated the unique and combined effects of attachment to mothers and parent-rated conscientiousness with these emerging peer relationship profiles. Addressing these study associations in peer relationship profiles among early adolescents from low-income families would gain essential insights into professional knowledge fostering those vulnerable youth’s social adjustment and integration into societies. Based on the current literature review, we generated the following hypotheses:

**Hypothesis 1.**  *Four peer relationship profiles characterized by distinctive levels of peer acceptance and friendships may be empirically derived: socially competent, socially accepted, isolated, and rejected*.

**Hypothesis 2.**  *Adolescents with secure attachment to mothers would be more likely to have group memberships of the socially competent or socially accepted profiles than the isolated or rejected profiles (main effect; 2a). The positive association between secure attachment to mothers and having group memberships of the socially competent or socially accepted profiles might be pronounced for adolescents with higher levels of conscientiousness (versus lower levels of conscientiousness; additive interaction effect; 2b). Alternatively, such a positive association might be heightened for adolescents with lower levels of conscientiousness (versus higher levels of conscientiousness; compensatory interaction effect; 2c)*.

## 2. Method

### 2.1. Participants and Procedures

The current investigation was based on a larger research project focusing on exploring the correlates of positive youth development. The measures used in the present study stand for a subset of the questionnaires administered within this larger project, which also consisted of other contextual (e.g., parenting styles and teacher-student relationships) and personal variables (e.g., Dark Triads and grit). Prior to this investigation, research materials and objectives were ethically reviewed and approved by the departmental ethics committee affiliated with Wenzhou University. After obtaining ethics approval, we obtained permission from school principals and head teachers in each collaborating school (located in North mainland China—Harbin and cities nearby). Head teachers sent a QR code to the parent-teacher Wechat group (a popular social media platform in China) with a brief description of this project, together with parental consent forms and parent-report measures. Parents or guardians were asked to read the consent forms carefully; if agreed, they could submit the online-based consent forms with electronic signatures. Those adolescents who returned their parental consent forms were asked to assent to the participation of this study. Adolescents’ self-reported assessments and peer nomination procedures were organized by group administration under the supervision of trained research assistants and head teachers in each classroom during school hours. Parents or guardians and eligible adolescents were all informed that participation in this investigation was entirely voluntary and confidential, and that data obtained from them could be eliminated during any research processes. Overall, the response rate in the current investigation was more than 90%, which was in line with prior research on Chinese adolescents [48,49].

A convenience sample of 1150 adolescents nested in 39 classrooms participated in the current investigation. The eligibility criteria of the participants in this study were as follows: (a) adolescents aged between 10 and 13 years old; (b) adolescents had a permanent residence in an urban context; and (c) adolescents’ families were registered in the governmental allowance system for low-income families. A detailed illustration of data exclusion can be found in the Supplementary Materials (Figure S1). After applying these criteria and excluding adolescents with high missing values, a final sample of 295 early adolescents (42.7% girls) from low-income urban families aged between 10 and 13 (*M_age_* = 10.94; *SD* = 0.80) was involved in this study. The sociodemographic characteristics of the sample are shown in Table 1.

### 2.2. Measures

#### 2.2.1. Peer Acceptance

Peer acceptance (group level of peer relationships) was assessed, using sociometric nominations, to have a higher level of objectivity than, for instance, self-reports. Peer nominations were collected following the procedures described by prior empirical studies on adolescents [48,50]. Specifically, we asked students one single item question “who in your classroom do you like the most?” This single item was administrated intentionally to decrease young adolescents’ participation burden. In company with this question, we provided a list of all classmates and asked students to nominate up to three classmates with whom he/she most liked to interact or play, irrespective of their classmates’ gender. For each student, the number of being nominated by other students was first summarized, and further standardized within each classroom to allow for appropriate comparisons. Of note, the excluded participants can also provide nominations when assessing peer acceptance, and the standardized scores were calculated based on the full sample instead of the selected sample. In this regard, higher values indicate higher levels of peer acceptance in youth own classrooms.

#### 2.2.2. Friendships

Friendships (dyadic level of peer relationships) was measured using the 6-item subscale of the Children’s Loneliness and Social Dissatisfaction Scale [51]. We selected this validated subscale to measure friendships because prior research has suggested that a friendship during early adolescence ascribes special significance to feelings of loneliness and isolation [52,53]. One item example is, “I have a lot of reciprocated friendships.” Items were rated on a 1 (*not true at all*) to 5 (*always true*) scale. The average score of these items was computed, with higher values representing greater self-perception of friendships. Cronbach’s alpha was 0.87 in the current sample.

#### 2.2.3. Attachment to Mothers

Attachment to mothers was assessed using the subscale of the Inventory of Parent and Peer Attachment (IPPA) [54]. The IPPA has been validated and extensively employed in Chinese youth studies [55]. The subscale employed consists of 25 items and three dimensions (i.e., trust, communication, and alienation). One of the sample items is, “I share thoughts and feelings with my mother (communication dimension).” The IPPA employs a 5-point Likert scale ranging from 1 (*never true*) to 5 (*always true*), and the average scores of all three dimensions were computed, with alienation scores reversed. A higher value, in this regard, represented a higher quality of the attachment relationships with the mother. In the present sample, Cronbach’s alpha was 0.85.

#### 2.2.4. Conscientiousness

Conscientiousness was measured by one of the subscales of the Big Five Inventory (BFI-44) [39]. This Chinese-validated subscale consists of nine items. For the purposes of this study, we changed item wordings to suit parent reports by changing statements starting with “I am …” to “My son/daughter …” One of the item examples is, “my son/daughter does a thorough job.” Either mothers or fathers rated each item on a 5-point scale ranging from 1 (*completely disagree*) to 5 (*completely agree*). No parents’ gender differences in the reports on conscientiousness were discovered. The average score of all items was calculated, with higher scores indicating higher levels of conscientiousness. In the present study, the subscale employed also demonstrated good internal consistency (Cronbach’s alpha = 0.80).

#### 2.2.5. Covariates

The present research controlled for a few sociodemographic characteristics, including age, gender, and family socioeconomic status [56]. Family socioeconomic status was computed by a standardized composite score of educational background, occupational status, and family income, following prior research [3]. Higher values, in this study, indicated higher levels of family socioeconomic status.

### 2.3. Data Analyses

Data analyses were conducted using SPSS 28.0 [57] and Mplus 8.3 [58]. Twenty adolescents were excluded due to high rates of missing data (more than 50%) in at least one of the questionnaires in this study. For instance, for the measure of friendships, if the participants missed more than three items, those participants were filtered out from the dataset directly. The remaining missing values were first evaluated by a Little’s Missing Completely at Random (MCAR) test and then imputed based on the expectation-maximization algorithm [59]. Prior to addressing research aims, a descriptive analysis of the study variables (means and standard deviations) was performed. At the same time, bivariate correlations among study variables were computed to have a preliminary overview of their associations.

To address the first hypothesis, we conducted a latent profile analysis. Latent profile analysis is a statistical method that identifies groups of participants who represent the highest intra-class invariances on the same set of observed indicators within a given group and the highest variances between subgroups [60,61]. We started to explore peer relationship profiles with a one-profile model and systematically increased the number of profiles until a five-profile solution. An optimal model fit was selected and inspected according to conventional criteria for fit indices (AIC, BIC, and aBIC) and relative fit indices, such as the likelihood ratio tests (LMR-LRT and BLRT values) [62,63]. The likelihood ratio tests should be significant, indicating that the given model with *k* profiles is superior to the model with *k*-1 profiles. At the same time, classification accuracy (entropy), the smallest number of the profiles, and profiles’ interpretability were also considered.

To address the second hypothesis, we performed a multiple multinomial logistic regression with the profile memberships (a categorical variable) as the outcome variable. The current data set had a nested design, in which students were recruited from 39 classrooms, but the initial estimation regarding the interclass coefficients across different classrooms exhibited low variances in peer-nominated acceptance and self-reported friendships. For simplicity, we did not consider employing a multilevel analysis thereafter. The study variables (attachment to mothers and conscientiousness), two-way interaction terms between the study variables, and confounding sociodemographic variables (i.e., age, gender, and family socioeconomic status) were entered into the model simultaneously. Post hoc simple slope analyses were conducted, adhering to the standards suggested by Aiken and West (1991) [64], to estimate the specific nature of significant two-way interactions. Figures generated from simple slope analyses were subsequently visualized to aid in the interpretation of interaction effects.

## 3. Results

### 3.1. Descriptive Statistics

Table 2 summarizes the means and standard deviations for study variables and their bivariate correlations. As shown in Table 2, a moderate correlation between the scores for peer-nominated acceptance and friendships indicated good construct validity for the single peer nomination item. Additionally, peer-nominated acceptance and self-reported friendships were each positively related to attachment to mothers and parent-rated conscientiousness.

### 3.2. Identification of Peer Relationship Profiles

Table 3 displays the model fit indices of latent profile analysis. As shown in Table 3, the likelihood ratio tests (i.e., LMR-LRT and BLRT values) were significant until the three-profile solution, indicating that the model with a three-profile solution was better than a two-profile solution. The following four and five-profile solutions were not taken into account because of insignificant likelihood ratio tests. Moreover, the three-profile solution demonstrated good classification accuracy, as evidenced by the entropy value, and the smallest number of the three-profile solution was also appropriate. We therefore proposed a three-profile as an optimal model in this study. These emerging profiles were significantly differentiated across peer acceptance (*F* = 270.33, *p* < 0.001; Partial η^2^ = 0.64) and friendships scores (*F* = 241.92, *p* < 0.001; Partial η^2^ = 0.62), as shown by the MANOVA results (see Supplementary Materials Table S1 for mean differences across three peer relationship profiles).

**Figure 1:**
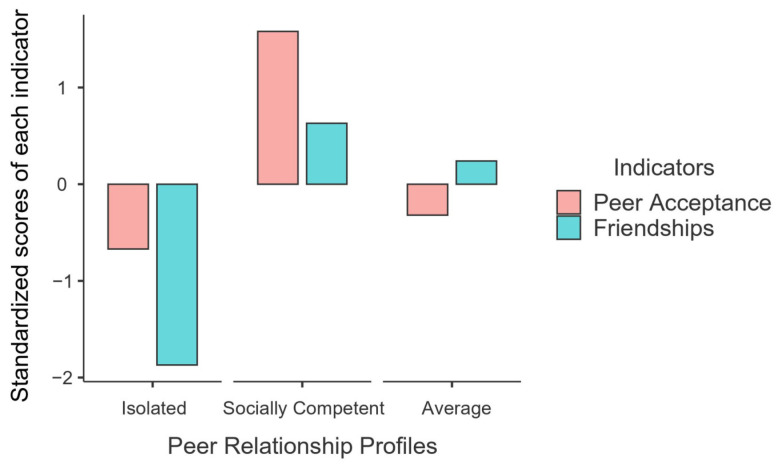
Three peer relationship profiles based on the standardized scores of peer-nominated acceptance and self-reported friendships. Note. *N* = 295.

Figure 1 depicts the distribution of these three peer relationship profiles based on standardized scores of peer acceptance and friendships. Adolescents in the first profile (*n* = 43; 14.6%) reported below-average scores on both peer acceptance and friendships; thus, this profile was labeled “isolated”; adolescents in the second profile (*n* = 48; 16.3%) reported above-average scores on both peer acceptance and friendships; hence, this profile was labeled “socially competent”; adolescents in the third profile (*n* = 204; 69.1%) held on-average peer acceptance and friendship scores; thus, this profile was labeled “average”. The identification of the isolated and socially competent profiles supported the first hypothesis, but the emergence of the average profile was unexpected.

### 3.3. Unique and Combined Associations of Attachment to Mothers and Conscientiousness with Peer Relationship Profiles

Table 4 summarizes the statistical parameters of the multiple multinomial analysis, with the isolated profile being the reference group. As shown in Table 4, attachment to mothers and conscientiousness, both independently and interactively, were positively related to the profiles contrast between the isolated profile and the socially competent profile, and between the isolated profile and the rejected profile.

First, the main effects showed that adolescents with secure attachment to mothers were more likely to be group members of the socially competent or the average profiles than the isolated profile, supporting the main effect hypothesis (2a).

**Figure 2:**
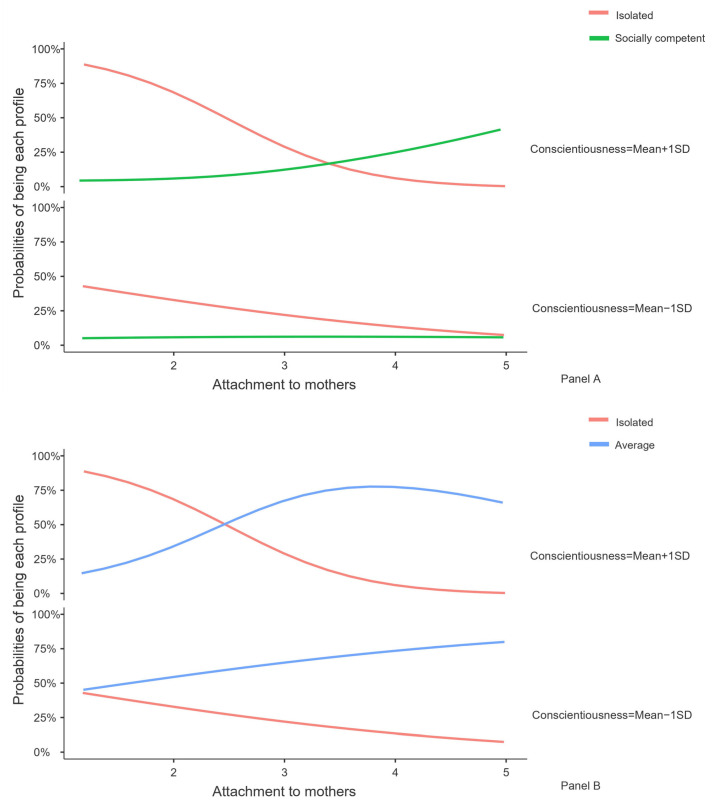
Plotting the interactions of attachment to mothers and conscientiousness on peer relationship profiles. *Note*. *N* = 295. Conscientiousness was divided into two levels based on mean + 1*SD* and mean − 1*SD*. The panels A and B in the figure refer to the label of the subfigures.

The significant interaction terms were subsequently probed by simple slope analyses and visualized figures. Figure 2 (Panel A) presents the profile contrast between the isolated profile and the socially competent profile. Simple slope analyses showed that this profile contrast was significant at higher (*M* + 1*SD*) levels of conscientiousness (*b* = 2.42, *SE* = 0.57, odds ratio = 4.18, *p* < 0.001), but not at the lower (*M* − 1*SD*) levels of conscientiousness (*b* = 0.40, *SE* = 0.47, odds ratio = 0.84, *p* = 0.39). Hence, we limited our interpretations to higher levels of conscientiousness. From a descriptive perspective, as the scores of attachment to mothers increased, the possibility of being group members of the isolated profile decreased, whereas the possibility of being group members of the socially competent profile increased. This interaction pattern supported the additive effect (2b).

Figure 2 (Panel B) presents the profile contrast between the isolated profile and the average profile. Simple slope analysis exhibited that this profile contrast was significant at the higher levels of conscientiousness (*b* = 1.63, *SE* = 0.44, odds ratio = 3.68, *p* < 0.001), but not at lower levels of conscientiousness (*b* = 0.53, *SE* = 0.30, odds ratio = 1.76, *p* = 0.07). Therefore, for adolescents who reported higher levels of conscientiousness, the interaction pattern between attachment to mothers and conscientiousness was further interpreted in Figure 2 (Panel B). As the scores of attachment to mothers increased, the possibility of being group members of the isolated profile decreased. In contrast, the possibility of being group members of the average profile increased, although after certain points, the possibility of being the average profile slightly dropped. This pattern again supported the additive interaction effect (2b).

## 4. Discussion

Despite dramatic economic growth in the past few decades, many youths particularly in developing countries continue to live in households with inadequate economic resources and have restricted access to public health services and educational resources. Elucidating the environmental and personal correlates of peer relationship profiles among early adolescents from low-income families is essential not only to facilitating youth optimal social adjustment and integration but also to potentially minimizing psychological costs triggered by economic disparity and maintaining public order and stability. Using research data gathered from multiple sources, the present study sought to extend the existing literature by exploring positive aspects of peer relationship profiles in a person-centered approach among early adolescents from low-income urban families. The current study also extended prior scholarship by delineating the unique and combined relations of attachment to mothers and conscientiousness with emerging peer relationship profiles among early adolescents from low-income urban families. Below, we discuss how these aims were met via a discussion of the findings.

The first aim of the current study was to explore peer relationship profiles among early adolescents from low-income urban families. The current study is among the first to distinguish the different combinations of peer acceptance and friendships toward a comprehensive understanding of the peer relationship patterns among early adolescents from low-income families. The current findings partially supported the first hypothesis, exhibiting three peer relationship profiles: isolated, socially competent, and average. Most adolescents from low-income families belonged to the average profile, characterized by on-average peer acceptance and friendship scores. One possible explanation for such a profile is distinct constellations of the risks triggered by familial poverty and resources provided by other social agents or contexts during early adolescence [5,6]. Thus, the majority of adolescents from low-income family backgrounds are able to sustain proper levels of social interactions with their peers. Another interpretation may be attributed to cultural emphases on modesty and dialectic thinking in Chinese societies. Dialectic thinking, for instance, significantly influences individuals’ manifestations of behaviors and emotions [65]. Under such belief, individuals tend to behave in a compromised manner and avoid extremities.

Subsequently, approximately 16% of adolescents from low-income families belong to a socially competent group, a profile characterized by a high level of peer acceptance and friendships. This profile aligns with the peer relationship profiles identified in prior research [19]. This alignment indicates that some adolescents can overcome family adversities and develop resilience by having positive social relations with peers. Finally, the remaining adolescents belonged to the isolated group, a profile characterized by congruently low scores on peer acceptance and friendships. This profile was also identified in Chen et al.’s (2019) research, and educators and practitioners should pay attention to this socially maladjusted group.

The second aim of this study was to examine the unique and combined associations of attachment to mothers and conscientiousness with the identified peer relationship profiles in early adolescents from low-income families. The findings supported the first hypothesis (the main effect; 2a), showing that adolescents with secure attachment to mothers were more likely to be group members of the socially competent and average profiles than the isolated profile. The findings generally supported the spillover and attachment theories, showing that the quality of the mother-child attachment affects adolescents’ cognitive representations of relationships, which help shape their future social interactions [20,29]. Accordingly, interactions with accessible and supportive mothers could positively influence how adolescents further interpret social signals and interact with peers [20,21,66].

Further, the current findings supported the additive interaction effect (2b) between attachment to mothers and conscientiousness on peer relationship profiles. Adolescents with secure attachment to mothers and high conscientiousness are more likely to effectively manage their emotions and behaviors and establish positive peer interactions. This may be because conscientious adolescents are more likely to establish close relationships with their mothers and are generally more aware of others’ support [37,38]. The positive association between secure attachment to mothers and positive peer relationships in this regard would be stronger for adolescents with higher conscientiousness (versus those with lower conscientiousness), because conscientious adolescents may better carry the positive aspects of mother-child relationships to build and maintain positive peer relationships [37,38].

The current study has several conceptual and methodological strengths. Conceptually, the current study advances prior scholarship by delineating the additive interaction pattern of attachment to mothers and conscientiousness on positive aspects of peer relationships. The findings generated facilitate our systematic understanding of the multi-layered influences in peer relationships, broadening both the spillover and attachment theories in low-income family contexts. Additionally, studying such associations in the “storm and stress” life period—early adolescence—not only fulfills the crucial gap in the literature but also delivers a developmentally meaningful message to the youth with low-income family backgrounds. At methodological and statistical levels, the current study gathers data from multiple perspectives and overcomes the limitations of prior research extensively concerned by common method bias. In addition, this study innovatively extends prior scholarship by exploring positive peer relationship profiles in a person-centered approach. Using such an approach, we empirically derive three naturally occurring subgroups that differ in both specific values on peer-nominated acceptance and friendship and correlate them with attachment to mothers and conscientiousness.

Along with these strengths, the results should be interpreted in light of the following limitations. First, a cross-sectional design of the current study does not allow researchers to establish the causality between attachment to mothers and adolescents’ peer relationships. Although much of the research regards parental attachment as a predictor of children’s social relationships, socialization processes within the peer group could impact early adolescents’ perceptions concerning relationships with attachment figures [67]. Future studies should therefore employ a longitudinal design to capture such dynamics and promote the understanding of different interpersonal relationships of young adolescents. Second, the current study only focused on a limited number of the variables within the broad ecological framework, although studying the roles of attachment to mothers and conscientiousness is conceptually important and practically meaningful. Future studies may extend the current findings by exploring other factors embedded in the systematic framework (e.g., attachment to fathers) ecological model that could affect the associations the present research tested.

### Theoretical and Practical Implications

These limitations notwithstanding, the current research conveys important theoretical and practical implications. Theoretically, examining a person-centered perspective of positive aspects of peer relationships can enhance our conceptual understanding of interpersonal and positive youth development theories. Positive aspects of peer relationships can be conceptually constructed by the distinct group- and dyadic-level indicators, characterized by distinct levels of peer acceptance and friendships. The present study broadens the spillover and attachment theories in a vulnerable family context (i.e., low-income family), delineating the unique and combined effects of attachment to mothers and conscientiousness on positive aspects of peer relationships.

At a practical level, school educators or practitioners working with adolescents from low-income families should assess the quality of mother-child relationships in order to develop a better understanding of contextual support that may contribute to positive peer relationships. The current findings also affirm the importance of including conscientiousness in future intervention studies, enhancing intervention efficacy [68]. In this regard, educators or practitioners can better locate adolescents from low-income families for future intervention or prevention programs. For instance, for those with low levels of conscientiousness, educators or practitioners should organize some activities to improve their levels of conscientiousness [69].

## 5. Conclusions

The current study integrates multiple data sources—such as the strength of the sociometric ratings, self-reports, and parent-rated evaluations—to potentially reduce shared method variance and provide a person-oriented and positive aspect of peer relationship patterns and their environmental and personal correlates among early adolescents from low-income urban families. The current study leverages a person-centered approach to delineate three naturally occurring, conceptually rich peer relationship patterns: isolated, socially competent, and average. Based on that approach, the results indicate that most early adolescents from low-income families are in the average profile. Further, the current findings broaden our understanding of the unique and combined associations that peer relationship patterns have with attachment to mothers and conscientiousness among early adolescents from low-income urban contexts. The findings also underscore why it is important to not only establish supportive social interactions between children and mothers, but also consider any individual differences when designing school-based personalizing intervention or prevention programs.

## Figures and Tables

**Table 1:** Sociodemographic characteristics of the sample.

	*N*	%
**Age (M ± SD years)**	10.94 ± 0.79	
**Gender (girls)**	126	42.7%
**Parental education (mother/father)**		
Middle school or lower	169/143	57.3%/48.5%
High school	105/129	35.6%/43.7%
University or college education	20/19	6.8%/6.4%
Master’s degree or higher	1/4	0.3%/1.4%
**Income (Chinese Yuan)**		
Less than 3000	69	23.4%
3000–5000	226	76.6%

**Table 2:** Descriptive statistics and bivariate correlations.

Variable	*M*	*SD*	Range	1	2	3	4	5	6	7
1. Peer-nominated acceptance	−0.06	0.92	−1.47–3.55	—						
2. Self-reported friendships	4.12	0.86	1–5	0.38 ***	—					
3. Self-reported attachment to mothers	4.00	0.68	1–5	0.15 *	0.37 ***	—				
4. Parent-rated conscientiousness	3.44	0.89	1–5	0.19 **	0.24 ***	0.17 **	—			
5. Age	10.94	0.79	10–13	−0.02	−0.01	−0.12 *	0.04	—		
6. Gender ^a^	-	-	1–2	0.07	0.17 **	0.05	0.17 **	−0.07	—	
7. Socioeconomic status	−3.92	2.15	−8.99–3.22	0.07	0.01	0.05	0.01	−0.08	0.07	—

*Note. N* = 295. ^a^ coded as 0 = boys, 1 = girls. * *p* < 0.05, ** *p* < 0.01, *** *p* < 0.001.

**Table 3:** Goodness of fit indices for different latent profile solutions.

	AIC	BIC	aBIC	Entropy	ALMR LRT	BLRT	Smallest Profiles (%)
1-Profile	1631.58	1646.33	1633.64	-	-	-	-
2-Profile	1551.17	1576.98	1554.78	0.84	81.62 **	86.40 **	19.3%
**3-Profile**	**1480.79**	**1517.66**	**1485.95**	**0.82**	72.15 **	76.37 **	**15.0%**
4-Profile	1452.68	1500.61	1459.39	0.81	32.22	34.11	5.0%
5-Profile	1440.60	1499.59	1448.85	0.80	17.08	18.08	4.2%

*Note*. *N* = 295. The optimal model is highlighted in bold type. ** *p* < 0.01.

**Table 4:** Regression analysis predicting peer relationships profiles from direct and interactive associations of attachment to mothers and conscientiousness.

Profile Contrast	Variables	*b*	*SE b*	95% CI for *b*		Odds Ratio	*p*
Isolated vs. Socially Competent	Attachment to mothers	1.42	0.38	0.67	2.16	4.12	<0.001
	Conscientiousness	0.96	0.29	0.39	1.53	2.61	<0.001
	Age	0.03	0.29	−0.55	0.60	1.03	0.931
	Gender ^a^	0.44	0.48	−0.51	1.39	1.55	0.363
	Socioeconomic status	0.02	0.10	−0.18	0.22	1.02	0.819
	Attachment to mothers X Conscientiousness	1.13	0.41	0.31	1.94	3.08	0.007
Isolated vs. Average	Attachment to mothers	1.08	0.27	0.56	1.61	2.96	<0.001
	Conscientiousness	0.50	0.22	0.07	0.94	1.66	0.024
	Age	0.19	0.23	−0.25	0.64	1.21	0.394
	Gender ^a^	0.52	0.39	−0.24	1.28	1.68	0.181
	Socioeconomic status	−0.09	0.08	−0.25	0.07	0.91	0.271
	Attachment to mothers X Conscientiousness	0.61	0.30	0.02	1.20	1.85	0.041

*Note*. *N =* 295. ^a^ coded as 0 = boys, 1 = girls.

## Data Availability

The data presented in this study are available on request from the first or corresponding author.

## Supplementary Materials

The following supporting information can be downloaded at: https://www.mdpi.com/article/10.3390/ijerph20054349/s1, Table S1: Mean differences in peer acceptance and friendships across three peer relationship profiles. Figure S1: Data exclusion flowchart.
