# Supplementary material for: Peer Relationship Profiles among Early Adolescents from Low-Income Families: The Unique and Combined Effects of Attachment to Mothers and Conscientiousness
Source: Int J Environ Res Public Health. 2023 Feb 28;20(5):4349. doi: 10.3390/ijerph20054349 (PMC10002007; doi:10.3390/ijerph20054349)

Table S1

Mean differences in peer acceptance and friendships across three peer relationship profiles

	1.Isolated (n = 43)		2.Socially Competent (n = 48)		3.Average (n = 204)				
	M	*SD*	*M*	*SD*	*M*	*SD*	*F*	Partial η^2	Post hoc
Peer-nominated acceptance	-0.67	0.47	1.58	0.63	-0.32	0.53	270.33^{***}	0.64	2 > 3 > 1
Self-reported Friendships	2.50	0.62	4.66	0.42	4.33	0.52	241.92^{***}	0.62	2 > 3 > 1

Note. N = 295. *** $p < .001$

Figure S1

Data exclusion flowchart

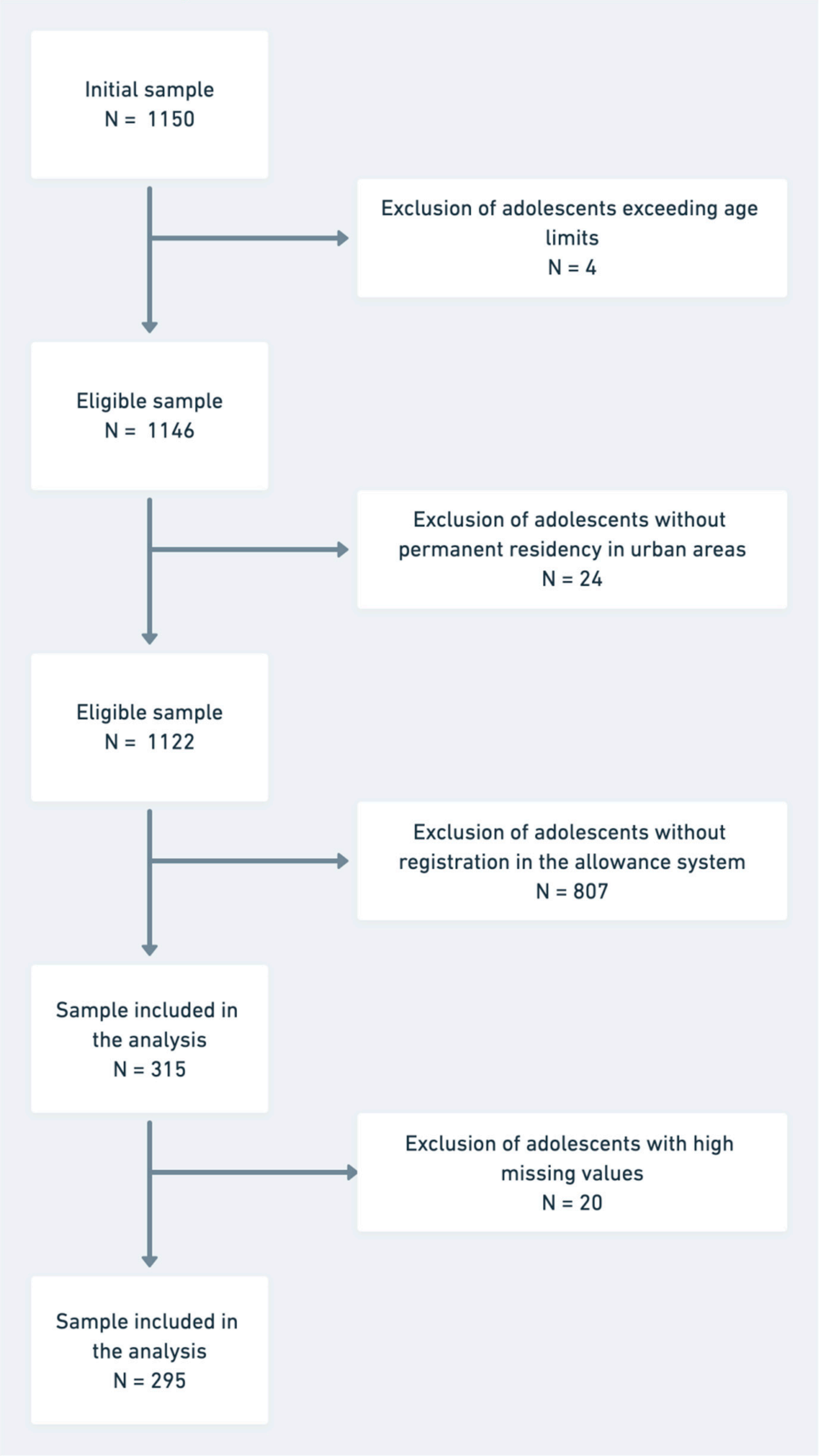

Supplement: Supplementary file 1 [file ijerph-20-04349-s001.zip › ijerph-2198154-supplementary.pdf]
